# DNA Hydroxymethylation in Smoking-Associated Cancers

**DOI:** 10.3390/ijms23052657

**Published:** 2022-02-28

**Authors:** Ahmad Besaratinia, Amanda Caceres, Stella Tommasi

**Affiliations:** Department of Population & Public Health Sciences, USC Keck School of Medicine, University of Southern California, M/C 9603, Los Angeles, CA 90033, USA; Amanda.Caceres@med.usc.edu (A.C.); tommasi@med.usc.edu (S.T.)

**Keywords:** 5-hydroxymethylcytosine (5-hmC), cancer, demethylation, isocitrate dehydrogenase (IDH), smoking, ten-eleven translocation (TET), tobacco, transcription

## Abstract

5-hydroxymethylcytosine (5-hmC) was first detected in mammalian DNA five decades ago. However, it did not take center stage in the field of epigenetics until 2009, when ten-eleven translocation 1 (TET1) was found to oxidize 5-methylcytosine to 5-hmC, thus offering a long-awaited mechanism for active DNA demethylation. Since then, a remarkable body of research has implicated DNA hydroxymethylation in pluripotency, differentiation, neural system development, aging, and pathogenesis of numerous diseases, especially cancer. Here, we focus on DNA hydroxymethylation in smoking-associated carcinogenesis to highlight the diagnostic, therapeutic, and prognostic potentials of this epigenetic mark. We describe the significance of 5-hmC in DNA demethylation, the importance of substrates and cofactors in TET-mediated DNA hydroxymethylation, the regulation of *TETs* and related genes (isocitrate dehydrogenases, fumarate hydratase, and succinate dehydrogenase), the cell-type dependency and genomic distribution of 5-hmC, and the functional role of 5-hmC in the epigenetic regulation of transcription. We showcase examples of studies on three major smoking-associated cancers, including lung, bladder, and colorectal cancers, to summarize the current state of knowledge, outstanding questions, and future direction in the field.

## 1. Introduction

An estimated 1.8 million new cancer cases were diagnosed in the United States in 2020 [1,2]. Cancer is the second leading cause of death in the United States, with an estimated toll of 606,520 American lives in 2020 [2,3]. A strong and substantial body of evidence indicates that the cause of many types of human cancer is tobacco use [4,5]. According to the International Agency for Research on Cancer (IARC), tobacco smoke contains nearly 70 carcinogens [6]. Although the percentage of adult cigarette smokers in the United States has declined from 42% in 1965 to 14% in 2018 [2,4], there are still 34 million Americans who are current users of tobacco cigarettes [5,7,8]. Cigarette smoking alone is responsible for approximately 30% of all cancer deaths in the United States [1,3]. The U.S. Surgeon General’s Report lists 15 types of cancer linked to cigarette smoking, including lung, bladder, bowel (colorectal), liver, pancreas, kidney and ureter (renal), stomach, cervix and ovary, esophagus, and head and neck (mouth, throat, nose, and sinuses) cancers [4].

While many cancers are caused by smoking, not all smokers will develop cancer [1]. A wide range of genetic, epigenetic, and immunological determinants underlie cancer initiation and progression [9,10,11,12,13,14]. Of these, epigenetic mechanisms have come to the forefront of cancer research, owing to their ability to initiate the disease as well as modulate its clinical course and progression [10,15,16]. As such, epigenetic modifications have shown utility for early diagnosis and monitoring of the progression of cancer [16,17]. Given the reversibility of epigenetic changes, they have also shown promise as targets for therapy and prognosticators of response or resistance to treatment [18,19]. Whilst the focus of this review is on DNA hydroxymethylation as a prime epigenetic modification with known and emerging roles in smoking-associated carcinogenesis [20,21,22,23,24], we refer the readers to comprehensive reviews on other epigenetic alterations, including DNA methylation, histone modifications and variants, microRNA (miRNA) dysregulation, chromatin remodeling, and nucleosome positioning [16,25,26,27,28,29,30]. Given the extensive body of research on DNA hydroxymethylation and smoking-associated cancers, we have highlighted examples of studies on lung, bladder, and colorectal cancers to summarize the current state of knowledge, outstanding questions, and future directions in this ever-evolving field.

### 1.1. DNA Hydroxymethylation: The Rebirth of an Old Cytosine Modification

5-hydroxymethylcytosine (5-hmC) was first detected in mammalian DNA in 1972 [31]. Research into 5-hmC, however, did not intensify until 2009 [32,33] when ten-eleven translocation 1 (TET1) was found to oxidize 5-methylcytosine (5-mC) to form 5-hmC and its derivatives [34,35]. The renewed interest in 5-hmC, which is dubbed the sixth DNA base [36] (5-mC being considered the fifth base [37]), has led to a wealth of information on the role of DNA hydroxymethylation in a wide range of biological functions, from DNA demethylation to pluripotency, cell differentiation, neural system development, aging, and pathogenesis of a large variety of diseases, especially cancer [21,22,24,33].

### 1.2. DNA Demethylation

**Figure 1:**
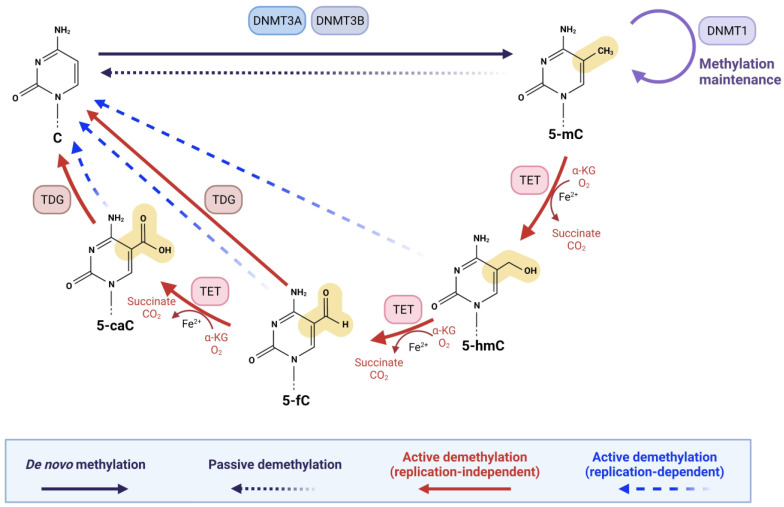
DNA demethylation in mammals. The two distinct mechanisms of erasure of 5-mC from the mammalian genome, also known as ‘DNA demethylation’, are shown. (1) In active demethylation, TET enzymes (TET1, TET2, and TET3) stepwise oxidize 5-mC to 5-hmC, 5-fC, and 5-caC. 5-fC and 5-caC are then excised by TDG and processed by BER, resulting in reversion of 5-mC to cytosine. Alternatively, replication-dependent dilution of 5-mC oxidation products takes place when 5-hmC, 5-fC, and 5-caC are reversed to unmodified cytosine during DNA replication (see Section 1.2 for detailed description). (2) In passive demethylation, failure of DNMTs function results in replacement of 5-mC by cytosine and dilution of 5-hmC content during DNA replication. 5-caC = 5-carboxylcytosin; 5-fC = 5-formylcytosine; 5-hmC = 5-hydroxymethylcytosine; 5-mC = 5-methylcytosine; α-KG = α-ketoglutarate; BER = base excision repair; C = cytosine; DNMT = DNA methyltransferase; TETs = ten-eleven translocation enzymes (TET1, TET2, and TET3); TDG = thymine DNA glycosylase. Figure was created using the software by BioRender (https://biorender.com/, accessed on 8 February 2022).

In mammals, DNA demethylation occurs through two distinct pathways: (I) ‘active’ demethylation and (II) ‘passive’ demethylation [21,24]. Active demethylation is achieved by TET enzymes whereby TET1, TET2, and TET3 sequentially oxidize 5-mC to 5-hmC, 5-formylcytosine (5-fC), and 5-carboxylcytosine (5-caC), followed by replication-dependent dilution or thymine DNA glycosylase (TDG)-dependent base excision repair (BER) [21,38]. As shown in Figure 1, 5-fC and 5-caC are excised by TDG enzyme and processed by BER, thus resulting in an unmodified cytosine [21,24,38]. Importantly, TDG is highly proficient in recognizing and removing 5-fC and 5-caC from the genome; however, it is highly inefficient in recognizing 5-hmC [39,40,41]. The substrate specificities of TETs and TDG ensure the stability and abundance of 5-hmC in the genome, supporting the view that 5-hmC is a persistent epigenetic mark [24,33]. Alternatively, replication-dependent dilution of the oxidation products of 5-mC takes place when 5-hmC, 5-fC, and 5-caC are reversed to unmodified cytosines during DNA replication, a demethylation process also known as ‘active modification–passive dilution’ (Figure 1) [17,21,42]. During DNA replication, unmodified cytosines are incorporated into the newly synthesized strand, creating hemi-modified CpG dinucleotides [43]. A 5-mC:C dyad is recognized by ubiquitin-like with PHD and ring finger domains 1 (UHRF1), which helps recruit DNA methyltransferase 1 (DNMT1) to the hemi-5 mC site [44,45,46]. This ensures faithful re-establishment of 5-mC on the newly synthesized strand after DNA replication [43]. It has been shown that UHRF1 has lower affinity for 5-hmC:C than 5-mC:C [47,48]. In addition, DNMT1 is much less efficient at 5-hmC:C, 5-fC:C, and 5-caC:C dyads than at a 5-mC:C dyad [47,48,49,50]. Through successive rounds of DNA replication, a 5-hmC-, 5-fC-, or 5-caC-modified CpG dinucleotide can become demethylated (Figure 1) [21]. 

As mentioned above, maintenance of DNA methylation is accomplished by DNMTs during DNA replication through methylation of hemimethylated CpG dinucleotides [43,51]. Failure of DNMTs in converting cytosine to 5-mC results in passive DNA demethylation [24,52]. It is suggested that deamination of 5-mC to thymine and possibly of 5-hmC to 5-hydroxymethyluracil catalyzed by deaminases, followed by excision repair with TDG, can also lead to the generation of an unmodified cytosine (Figure 1) [24,38,53].

## 2. TET-Mediated Oxidation of 5-mC

The TET family of enzymes, TET1, TET2, and TET3, in mammalian cells are α-ketoglutarate (α-KG)-dependent dioxygenases [21,32,54]. The α-KG-dependent dioxygenases are a major class of non-heme iron proteins, with nearly 70 members in mammals that catalyze diverse reactions, including hydroxylation, demethylation, ring open expansion, ring closure, and desaturation [55,56]. All TET enzymes utilize the same reaction mechanism and cofactors but act on a variety of substrates to catalyze the sequential oxidation of 5-mC to 5-hmC, 5-fC, and 5-caC [21,57,58]. The interest in TET proteins has primarily centered around the idea that 5-hmC, 5-fC, and 5-caC could serve as intermediates in DNA demethylation [20,21,22,23]. 5-fC and 5-caC are both removed by TDG coupled to BER, resulting in unmodified cytosines and therefore DNA demethylation (see, Figure 1) [21,39,40,42,59,60]. However, growing research also shows the additional significance of TET-mediated oxidation of 5-mC in a wide variety of biological functions and diseases, including various types of malignancies [23,24].

### 2.1. TETs Regulation: The Importance of Substrates and Cofactors Availability

Similar to other α-KG-dependent dioxygenases, TET enzymes require α-KG (also known as 2-oxoglutarate) and molecular oxygen as substrates and Fe^2+^ as a cofactor to convert 5-mC to 5-hmC, 5-fC, and 5 caC and generate CO_2_ and succinate [42,61,62]. Many of the TET enzymes also require a reducing agent, such as vitamin C, an essential antioxidant that reduces Fe^3+^ to Fe^2+^ [21]. Changes in cellular iron concentration have been shown to cause alteration in 5-hmC levels [63]. Moreover, mutations affecting the critical iron-binding residues of TETs are known to reduce the catalytic activity of these enzymes [64]. Vitamin C is known to stimulate the enzymatic activity of TETs, most likely through acting as a cofactor. Mechanistically, vitamin C directly interacts with the catalytic domain of TET proteins to enhance their enzymatic activity [65,66,67]. Additionally, vitamin C may promote TET folding to facilitate the recycling of Fe^2+^ [67].

The tricarboxylic acid cycle (otherwise known as the ‘Krebs cycle’), enzyme cytosolic isocitrate dehydrogenase (IDH1), and its mitochondrial homolog (IDH2) are responsible for generating α-KG, making them an integral part of the pathway involved in TET-mediated 5-hmC production [20,61]. *IDH1* and *IDH2* mutations are heterozygous, and the mutant isoforms generate 2-hydroxyglutarate (2-HG) instead of α-KG [20]. The accumulation of 2-HG in cells and tissues inhibits enzymes that require α-KG as a cosubstrate, including the TET proteins [68,69,70,71]. Overexpression of *IDH1* or *IDH2* promotes 5-hmC formation in cells [71,72]. Conversely, downregulation of *IDH2*, as observed in certain types of cancer, is associated with decreased levels of 5-hmC [72]. Moreover, buildup of succinate or fumarate, which are structurally similar to α-KG and 2-HG, has been shown to inhibit TETs and other α-KG-dependent dioxygenases [68], resulting in loss of 5-hmC [73,74]. Importantly, mutations in the tricarboxylic acid cycle enzymes succinate dehydrogenase (SDH) and fumarate hydratase (FH), which interconvert succinate and fumarate, respectively, are highly common in cancer [75,76]. Collectively, the availability of substrates and cofactors can directly affect the reaction kinetics of TETs [21]

### 2.2. Post-Transcriptional and Post-Translational Regulation of TETs

TET-mediated oxidation reactions can be modulated by post-transcriptional and post-translational regulation of TETs [21]. Following transcription, mRNAs of *TETs* [77,78,79,80,81,82,83,84,85,86,87] and *TDG* [82,83,84,88] can be regulated by miRNAs. Specifically, *TETs* and *TDG* are long genes and encode large proteins (180–230 kDa and 75–80 kDa, resp.), which can be targeted post-transcriptionally by miRNAs, particularly in cancer, where the dysregulation of diverse classes of miRNA is very common [20,21,88]. After translation, the subcellular localization, chromatin binding, and enzymatic activity of TET proteins can be regulated by covalent modifications, such as ubiquitylation [89], acetylation [90], phosphorylation [91], GlcNAcylation [92,93,94], and PARylation [95]. Protein levels of TETs can also be regulated by protein–protein interaction [96] and proteolysis [90,97].

## 3. Biological Significance of 5-hmC

TET enzymes have a lower affinity for 5-hmC when compared to 5-mC [21]. Consistent with the poor stability of 5-fC and 5-caC and their amenability to repair by BER, 5-hmC content in the genome is much higher (10- to 100-fold) than those of its oxidized derivatives [41,58,98,99,100]. In addition, 5-hmC has been detected at levels as high as 0.7% (of total nucleotides) in certain cell types, such as neuronal cells of the central nervous system (CNS) [34,101]. These findings indicate that 5-hmC is not always oxidized to yield derivatives removable by BER [21,38]. Thus, while 5-hmC is important for DNA demethylation [33,38], it may also have other biological functions [20,21,22,23].

### 3.1. Cell-Type Dependent Distribution of 5-hmC

Whereas global 5-mC content is relatively consistent across normal adult tissues (4–5% of all cytosines), 5-hmC content is moderately low (~0.4% of all cytosines and ~10% of all mC) and varies greatly across tissues (between 0.03 and 0.7%) [20,53,101,102]. The highest levels of 5-hmC are found in the adult brain, especially in the hypothalamus and in the cerebral cortex and other compartments [34,36]. Of significance, most neuronal cells in the adult brain have ceased to divide mitotically [103,104]. The varying abundance of 5-hmC across different cell types, with higher levels in post-mitotic cells (being highest in cells of the CNS) and lower to barely detectable levels in proliferating cells, has been demonstrated in various species, including humans and mice [32,34,105,106]. The inverse relationship between 5-hmC content and cell division rate is best exemplified in rapidly dividing cells (with the exception of embryonic stem cells (ESCs) [53,98,107]) or highly proliferating cancer cell lines and tumors wherein no or an extremely low level of 5-hmC is detectable [24,32,108].

### 3.2. 5-hmC and Gene Regulation

Accumulating data show a prominent role for 5-hmC in the epigenetic regulation of gene expression [21,33,53,109,110]. Whether 5-hmC increases or decreases gene transcription is mainly dependent on cell type and its genomic location [21,33,53,111]. Abundant 5-hmC is found in gene bodies of active genes, and TET1 is often enriched at the transcription start site (TSS) of genes with high CpG promoters that are occupied by bivalent histone marks, H3K4me3 for transcription activation and H3K27me3 for transcription repression [21,23]. Compelling evidence supports that 5-hmC and TET proteins regulate gene expression through modulating chromatin accessibility of the transcriptional machinery or by affecting repressor binding [21,24,38]. This is consistent with the observed high levels of 5-hmC within gene bodies, promoters, and transcription factor (TF) binding sites [105,112,113,114,115]. In addition, immunohistochemical analysis has shown that 5-hmC is often accumulated in regions marked by H3K4me2/3 [53,112,116,117]. However, the relationship among 5-hmC, TETs, and gene expression is highly complicated and not always straightforward [21,33,38,114,118,119]. For instance, actively transcribed genes show reduced 5-hmC content in their TSS regions, while silenced or lowly expressed genes show abundant 5-hmC at promoters [21,38,120]. 5-hmC content in gene bodies is positively correlated to gene expression in certain cell types, whereas the opposite is true in other cell types [21,38,106]. Interestingly, many 5-hmC peaks of ESCs are lost in neural progenitor cells (NPCs), concomitant with a global loss of 5-hmC in the latter cell type, which implies that DNA hydroxymethylation may play a role in the differentiation of ESCs to NPCs [106]. Of significance, *TET1* and *TET2* are highly expressed in ESCs, which is consistent with the abundance of 5-hmC in these cells [120].

Differences in cell type seem to be a main determinant of the complex relationship between 5-hmC and gene expression [21,38]. For example, despite the abundance of 5-hmC in actively transcribed genes, for a gene with similar expression level in various tissues, there may be a 20-fold change of 5-hmC on the gene body among different tissues [105]. Altogether, the existing data show highly complex and interrelated connections among 5-hmC, TETs, and transcription regulation [21,33,38,114,118,119].

### 3.3. Functional Role of 5-hmC in Transcription

5-hmC may be involved in regulating gene expression through effects on diverse regulatory elements and processes [24,33,116,121]. Distribution of 5-hmC is varied at enhancers, promoters, TSSs, gene bodies, 3′ UTRs, or intragenic regions, consistent with the modulation of 5-hmC patterns by histone modifications, binding proteins of epigenetic marks, and chromatin configuration during cell differentiation and specification [38,116,122,123,124]. 5-hmC may function as a cis element to promote or repress gene expression by binding to TFs, such as activators or repressors in regulatory regions of genes, or by interacting with histone marks to alter chromatin configuration to switch genes ‘on’ or ‘off’ [21,38]. 5-hmC accumulates at the TSS of genes whose promoters are occupied with bivalent histone marks [115,125] and at ‘poised’ and active enhancers marked with H3K4me1, H3K18ac, and H3K27ac [116,121]. Developmental genes have ‘bivalent domains’ in promoters and are transcriptionally ‘poised’ [126]. In pluripotent ESCs, ‘bivalent domains’ can poise genes with both activating (H3K4me3) and repressive (H3K27me3) marks, so that they can be swiftly activated or silenced, depending on the specific differentiation pathway that is taken [126]. The TET-mediated generation and distribution of 5-hmC, modulation of global 5-hmC/5-mC content, and reprogramming of de novo ‘bivalent histone code’ in CpG island promoters directly influence bivalent domains of the poised genes [127,128,129]. 

TET1 forms a complex with polycomb repressive complex 2 (PRC2) at H3K27me3 positive regions in the ‘bivalent’ state [130], and TET1 depletion impairs PRC2 binding to these targets [112,113,114,115,131]. 5-hmC is abundant in both repressed (bivalent, TET1/PRC2-cobound) and activated (TET1-only) genes [113], suggesting that 5-hmC plays a role in the machinery responsible for the pluripotency switch [38]. Furthermore, 5-hmC may also modulate alternative splicing to regulate transcription [21]. Of note, alternative splicing is a conserved way of diversifying the transcriptome and proteome, and transcripts from ~90% of genes undergo alternative splicing [132]. Importantly, 5-hmC is more abundant in constitutive exons than in alternatively spliced exons [133].

## 4. The Interplay of TETs, IDHs, and 5-hmC in Cancer

The role of TET enzymes in human cancer has been most extensively studied in hematopoietic malignancies [22,23,38]. Mutations in the *TET2* gene are frequent events in these types of cancer [134,135,136]. *TET2* mutations are found in 20% of acute myeloid leukemias, 20% of myeloproliferative neoplasms, 45% of chronic myelomonocytic leukemia, and 15% of T-cell lymphomas [22,23,38]. The studied mutations do not show a complete loss of TET2 function and are primarily missense mutations [137,138,139,140]. While *TET1* mutations are found at lower frequencies, *TET3* mutations are infrequent and have been shown to cause embryonic lethality in mice, suggesting that these mutations may not be tolerated in vivo [20,140,141]. We refer the interested readers to elegant reviews on the role of TET enzymes in the pathogenesis of hematopoietic cancers [142,143,144,145,146]. 

Since TET enzymes are α-KG-dependent, they rely on IDH enzymes, which catalyze the oxidative decarboxylation of isocitrate to α-KG [21,54]. Mutations in *IDH1* and *IDH2* occur frequently in human cancers [147,148,149]. These mutations are known to affect the active site of IDH enzymes, leading to neomorphic enzyme activity and resulting in the conversion of α-KG to 2-HG [148,149]. 2-HG can then function as a competitive inhibitor of α-KG-dependent dioxygenases, including TETs [68]. Similarly to 2-HG, fumarate and succinate, which are known to accumulate in cancer cells due to their deficiency in FH and SDH enzymes [75,76], can compete with α-KG to inhibit TETs [64,68]. The inhibition of TET enzymes by 2-HG has been shown to induce hypermethylation of CpG islands at the gene promoters in cancer and is thought to impact proper cell differentiation [147]. Changes in cellular state caused by *IDH* mutations may then promote malignant transformation [70,71,150,151,152].

Although mutations in the *TET* or *IDH* genes have been found in some malignancies, they are not present in all tumor types [21,148,149]. Solid tumors generally show global loss of 5-hmC [22,23,24]. Oxidative stress has been implicated as a likely event contributing to the global 5-hmC loss observed in human cancers [11,33,90,153]. Oxidative stress is thought to reduce global 5-hmC levels through effects on TET enzymes [33,154]. Of note, oxygen, in addition to α-KG, is a substrate of TET-mediated oxidation [61,62]. Recent findings have shown that oxidative stress leads to post-translation modification of TET2 and reduction of 5-hmC content [21,90]. 

It has also been demonstrated that hypoxia, a hallmark of cancer, can varyingly change 5-hmC levels in different cell types [21,24]. In response to hypoxia, certain cell types show increased levels of 5-hmC, an effect caused by a hypoxia-inducible factor (HIF)-mediated upregulation of TET [155,156,157]. In other cell types, however, hypoxia causes a reduction in 5-hmC levels without downregulating TET and independently of changes in reactive oxygen species (ROS) production, cell proliferation, and metabolite concentrations, suggesting a direct regulation by oxygen availability [21]. The association between hypoxia and 5-hmC loss is shown in tumor samples from glioblastoma patients and has been validated in a mouse breast tumor model [157]. Although the underlying mechanisms of modulation of 5-hmC levels in cancer remain to be fully elucidated, the existing data collectively support a key role for tumor hypoxia in DNA hydroxymethylation in human carcinogenesis [21,24,61,157].

## 5. Cigarette Smoking, Oxidative Stress, DNA Hydroxymethylation, and Cancer

Cigarette smoke contains several thousand chemicals of which many act as oxidants, pro-inflammatory agents, carcinogens, and tumor promoters [6]. Owing to their redox activity, various constituents of cigarette smoke can directly induce oxidative stress [158]. In addition, they can also trigger an inflammatory response, which can, in turn, cause oxidative stress [158,159,160]. Chronic inflammation and closely related oxidative stress are key components of tumorigenesis and directly linked to carcinogenesis [161,162,163,164,165,166,167,168]. Oxidative stress caused by cigarette smoking is thought to contribute significantly to the development of smoking-associated cancers (reviewed in ref. [167]).

An emerging area of interest is the role of DNA hydroxymethylation modulated by smoking-induced oxidative stress in the initiation and progression of cancer [167]. A recent study by our group has demonstrated significantly reduced global 5-hmC levels in the peripheral blood leukocytes of healthy smokers as compared to nonsmokers, matched for age, gender, and race [169]. The global 5-hmC levels in the study subjects were inversely and statistically significantly correlated to their smoking indices, including pack year and concentrations of plasma cotinine (a major metabolite of nicotine) [169]. 

While additional data are becoming available [170,171] and new research is underway to comprehensively determine the levels and genomic distribution of 5-hmC in healthy smokers, a wealth of information exists on the DNA hydroxymethylation status in smoking-associated cancers [23,24]. By leveraging the existing data on the quantification and mapping of 5-hmC in smoking-associated carcinogenesis, we can determine the potential of this epigenetic modification as a biomarker for the early detection of smoking-related cancers as well as a predictor of response or resistance to treatment. Identifying these mechanistic biomarkers and validating their sensitivity, specificity, and versatility in relevant (patient) populations will not only improve future strategies for diagnosis, treatment, and prognosis of smoking-associated malignancies, but it may also allow assessment of cancer risk in healthy smokers.

## 6. Lung Cancer

Lung cancer is the leading cause of cancer-related deaths worldwide [1,3]. In the United States alone, an estimated 228,820 new cases of lung cancer were diagnosed in 2020, with 135,720 deaths attributable to the disease [2]. The five-year survival rate for lung cancer is 19%. Only 16% of lung cancer cases are diagnosed at a localized stage when the survival rate is 57% [2]. This underscores the urgent need for identifying biomarkers of early detection for lung cancer [172,173,174]. Studies investigating the diagnostic utility of 5-hmC have found it to be a useful biomarker for the early detection of lung cancer. In addition, 5-hmC has shown promise as a good indicator of lung tumor stage, which is important considering that different stages of lung cancer require different therapies [173]. For example, early stage non-small-cell lung cancer (NSCLC) is treated surgically, whereas patients with locally advanced disease (stage III) require multimodal therapy [174,175]. Personalized treatment based on the patient’s tumor stage and clinical conditions has shown to improve the overall survival in lung cancer cases [176,177]. Smoking is the most dominant risk factor for lung cancer, with 80% of lung cancer deaths linked to smoking [3,4]. Because changes in the genomic distribution and levels of 5-hmC are common in the early stages of lung carcinogenesis—sometimes even prior to clinical manifestation of the disease [22,24,170]—this epigenetic modification may also be exploited as a biomarker for identifying smokers who are at increased risk of developing lung cancer. 

Several studies have investigated the genome-wide patterns of 5-hmC in tumor specimens from lung cancer patients (Table 1). Li et al. [178] have used the oxidative bisulfite sequencing (oxBS-seq) method to attain a single-base resolution of the hydroxymethylome in three pairs of human lung tumors and normal tissues. They have also used RNA-seq to determine the relationship between 5-hmC and gene expression. The authors reported detection of 5-hmC in promoters, gene bodies, and transcription termination regions, which showed strong positive correlation with gene expression and highly corresponded with H3K4me1 modification, a histone mark associated with active transcription. These findings support the utility of 5-hmC as a marker of active genes and a key determinant of gene expression in lung cancer [178].

Wang et al. [179] have analyzed the quantity and patterns of 5-hmC in eight lung squamous cell carcinoma (LUSC) tissues and adjacent normal tissues using TET-assisted bisulfite–Infinium Methylation EPIC BeadChip array. A global depletion of 5-hmC together with enrichment of hydroxymethylation in CpG islands and gene upstreams was detectable in tumor tissues as compared to controls. Gene set analysis revealed that the differentially hydroxymethylated genes were likely to converge at pathways involved in the cellular process, biological regulation, and metabolic process. Hierarchical clustering of the significantly differentially hydroxymethylated targets clearly distinguished tumor tissues from controls, supporting the discriminatory power of 5-hmC modeling for lung cancer diagnosis [179].

Two independent studies have quantified 5-hmC in the plasma cell-free DNA (cfDNA) of lung cancer patients as compared to controls. The first study by Song et al. [180] evaluated 5-hmC levels in the cfDNA of 15 lung cancer patients and eight healthy controls using a whole-genome 5-hmC sequencing method (hMe-Seal). Depletion of global 5-hmC was detectable in the cfDNA of lung cancer patients as compared to healthy controls. Among patients, the extent of reduction in global 5-hmC progressively increased as the disease advanced from early stage non-metastatic to late-stage metastatic lung cancer. These findings bear out the predictive value of 5-hmC for lung cancer staging as well as monitoring the progression of the disease. Song et al. also quantified 5-hmC in six other cancer types and found that the 5-hmC signature in lung cancer patients was distinct from not only healthy controls but also from patients diagnosed with other types of cancer [180]. 

The second study on the quantification of 5-hmC in the plasma cfDNA of lung cancer patients by Zhang et al. [181] employed the same hMe-Seal method to determine genome-wide distribution and levels of 5-hmC in 66 NSCLC patients and 67 healthy controls. The authors reported significant 5-hmC gain in both gene bodies and promoter regions in specimens from lung cancer patients as compared to healthy controls. It is important to note the geographic disparities in the studied populations by Zhang et al. and Song et al., as well as the varying sample sizes and heterogeneity of patients’ tumor stage in the respective reports [180,181]. Further research in larger and well-defined study populations is warranted to verify the use of 5-hmC detection in cfDNA as a minimally invasive tool for lung cancer diagnosis and prognosis.

Furthermore, mechanistic studies have investigated the role of TET enzymes in lung tumorigenesis. Forloni et al. [182] have reported that oncogenic epidermal growth factor receptor (EGFR) epigenetically silences diverse tumor suppressors in isogenic lung adenocarcinoma cell lines via transcriptional downregulation of *TET1* by the C/EBPα transcription factor. Of note, oncogenic EGFR is found in approximately 15% of lung adenocarcinomas and several other cancer types [183,184,185]. The authors demonstrated that the inhibition of oncogenic EGFR leads to binding of TET1 to tumor suppressor promoters and induces their re-expression through active DNA demethylation [182]. They also showed that ectopic expression of TET1 potently inhibits tumor growth in soft agar assays and significantly reduces tumor formation in athymic nude mice. In addition, small/short hairpin RNAs (shRNAs)-induced *TET1* knockdown confers resistance to EGFR inhibitors in lung cancer cells. Loss of *TET1* expression or mislocalized cytoplasmic TET1 is detectable in a substantial percentage of patient-derived lung cancer samples (~44%), suggesting that TET1 is likely inhibited in lung cancer [182]. Altogether, these results indicate that the disruption of *TET1* function can lead to the demethylation-dependent inactivation of tumor suppressor genes in lung cancer as well as adversely affect patients’ response to therapeutic options.

## 7. Bladder Cancer

In the United States, urinary bladder cancer is the fourth most common cancer in men, with an estimated 81,400 new cases and 17,980 deaths in 2020 [2]. The overwhelming majority of bladder cancer cases (~95%) are transitional cell carcinomas, which are primarily non-muscle invasive at the time of diagnosis and amenable to surgical treatment [186,187,188]. However, up to 80% of the treated cases will recur, of which 45% will progress to invasive cancer within five years [186,187,189]. The main screening method for bladder cancer is examination of the bladder wall with a cystoscope, which is both invasive and costly [187,190]. This method may also miss up to 30% of malignant cases [187,190]. Thus, there is a need for more accurate, efficient, and preferably non- or minimally-invasive methods to screen for bladder cancer [188,191,192,193]. Cigarette smoking is a major risk factor for bladder cancer [6,194]. Approximately half of all bladder cancer patients have a history of exposure to cigarette smoke [194]. Currently, the lag time between initial exposure to cigarette smoke and bladder cancer diagnosis is 20–30 years [1,3]. Studies investigating DNA hydroxymethylation in urological malignancies have found changes in the quantity and patterns of 5-hmC during early stages of these diseases (Table 1). Therefore, 5-hmC has the potential to be used as an epigenetic biomarker for the early detection of bladder cancer and a screening tool for identifying smokers who are at elevated risk of developing this malignancy.

Peng et al. [195] investigated the genome-wide patterns and levels of 5-hmC in matched bladder cancer and normal bladder tissues from 135 urothelial carcinoma patients as well as in bladder cancer cell lines and controls. Additionally, they evaluated the modulatory effects of vitamin C, a cofactor for TET enzymes, on DNA hydroxymethylation and inhibition of the malignant phenotype in both in vitro and in vivo models of bladder cancer. A global loss of 5-hmC was detected in bladder cancer tissues as compared to controls by both immunohistochemistry (IHC) and immunodot blot assays. Likewise, reduced global 5-hmC content was detectable in bladder cancer cell lines (T24, 5637, UMUC-3, and J82 cells) as compared to controls (Hum-u007: human normal bladder primary epithelial cells and SV-HUC-1: immortalized normal human urinary epithelial cells) by immunodot blot assay. Among bladder cancer patients, lower global 5-hmC levels correlated with higher tumor stage, lymphatic metastasis, and shorter overall survival, suggesting that the loss of 5-hmC is critical for bladder cancer progression, leading to poor clinical outcomes. Genome-wide mapping of 5-hmC was performed in paired bladder tumors and adjacent normal tissues using a hydroxymethylated DNA immunoprecipitation approach coupled with deep sequencing (hMeDIP-seq). A significant decrease in 5-hmC levels was observed within genes or in the regions 2 kb up- or downstream of the genes in bladder tumors as compared to control tissues. The differentially hydroxymethylated loci identified in bladder tumors mapped to 5,843 genes, with more than half of the targets being located either in exons (10.3%) or introns (58.9%) and 6.45% localizing to promoters. Kyoto Encyclopedia of Genes and Genomes (KEGG) pathway enrichment and gene ontology (GO) analyses revealed that the differentially hydroxymethylated genes in bladder tumors are closely associated with various cancer-related pathways. It was also shown that in vitro vitamin C treatment increases 5-hmC levels and inhibits malignant phenotypes in bladder cancer cell lines, as determined by cell proliferation analysis and apoptosis and colony formation assays. Similar results were found in vivo wherein intraperitoneal injection of vitamin C in a mouse xenograft model with human urinary bladder cancer T24 cells resulted in increased 5-hmC levels, reduced tumor growth, and decreased tumor burden [195]. 

Munari et al. [196] utilized an IHC staining method to evaluate global 5-hmC levels in tumor specimens and adjacent benign tissues from 55 patients with urothelial cell carcinoma of the bladder. A significant reduction in global 5-hmC levels was observed in urothelial cell carcinoma samples as compared to controls. No difference in global 5-hmC content was detectable between superficial and invasive lesions, suggesting that a loss of 5-hmC may represent an early event in bladder carcinogenesis. The global 5-hmC loss was not correlated with tumor grade or stage or the patients’ prognosis. However, the authors acknowledged that the study was not adequately powered to detect small prognostic differences based on 5-hmC levels because the number of progression and disease-specific death events were overall low [196]. The use of genome-wide sequencing-based approaches should facilitate the detection of site-specific changes in 5-hmC that may otherwise not be identifiable when using IHC analysis in small sample sizes.

The role of TET enzymes in bladder cancer progression and metastasis has been investigated in multiple studies. Zhu et al. [197] identified a new TET1/USP28/CD44/RhoGDIβ pathway, which is responsible for the oncogenic role of autophagy-related gene 7 (ATG7) in invasion, metastasis, and stem-like properties in human bladder cancer cells (T24T). Specifically, ATG7 overexpression inhibits AU-rich element RNA-binding protein 1 (AUF1) expression, which stabilizes *TET1* mRNA to increase its protein expression. Upregulated TET1 then directly demethylates the ubiquitin specific peptidase 28 (USP28) promoter, thereby enhancing USP28 transcription and expression. Binding of USP28 to CD44 standard (CD44s) protein leads to removal of the ubiquitin group from the ubiquitinated CD44s protein, resulting in the stabilization of CD44s protein to mediate the stem-like property of human bladder cancer cells. Furthermore, CD44s inhibits RhoGDIβ degradation, which in turn promotes human bladder cancer invasion and lung metastasis [197]. 

Hu et al. [198] reported a potential role of the X-inactive specific transcript (XIST)-TET1-p53 regulatory network in cell proliferation, migration, and apoptosis in bladder cancer. Importantly, *TP53* mutations are found in nearly half of all transitional cell carcinomas of the bladder, most frequently in high-grade invasive tumors [199]. The authors demonstrated that TET1 binds to the promoter region of the *TP53* tumor suppressor gene and promotes its expression in bladder cancer cells (T24), whereas XIST inhibits the expression of TP53 by binding to TET1. Knockdown of XIST significantly suppresses cell proliferation and migration and induces apoptosis in bladder cancer cells, whereas overexpression of XIST has the opposite effects. The TP53-mediated cell proliferation and migration and apoptosis in bladder cancer were suggested to be modulated by XIST-related inhibition of TET1 binding to *TP53* promoter, resulting in hypermethylation and decreased expression of this tumor suppressor gene [198]. 

## 8. Colorectal Cancer

Colon and rectal (colorectal) cancers are the third leading sites of new cancer and the second deadliest malignancy, with an estimated 1.9 million incidence cases and 0.9 million deaths worldwide in 2020 [190]. In the United States, 104,610 new cases of colon cancer and 43,340 new cases of rectal cancer, with 53,200 related deaths, have been estimated for the year 2020 [1]. The five-year survival rate for colorectal cancer is 64% [200,201]. The survival rate, when the cancer is at a localized stage, is 90%; however, only 39% of patients are diagnosed at this stage [200,201]. Additionally, both the incidence and death rates for colorectal cancer among adults younger than 55 are rising [2]. Currently, screening is recommended for people over the age of 45; however, the choice of colonoscopy, a routinely used and highly invasive screening method, makes this recommendation not widely adoptable [201,202]. The increase in cases among younger adults and lack of non-invasive screening tools highlight the need for novel biomarkers of early detection for colorectal cancer [202,203]. Over half of colorectal cancers in the United States are attributable to modifiable risk factors. Twelve percent of the incidence cases and 11% of the colorectal cancer deaths are directly linked to cigarette smoking [1,2]. Screening smokers to determine the ‘at risk’ individuals for colorectal cancer development may enable earlier detection of the disease, thus improving survival from this deadly malignancy. 

The roles of 5-hmC and TET enzymes in the initiation and progression of colorectal cancer have been investigated as a means to identify diagnostic and prognostic biomarkers for this disease (Table 1). Dziaman et al. [154] quantified 5-hmC levels in samples from colorectal cancer (CRC) patients (*n* = 97; paired tumors and normal colonic tissues), as well as patients with predisposing conditions, including benign polyps/colon adenomas (AD, *n* = 39) and inflammatory bowel disease (IBD, *n* = 49) [203,204,205]. In addition, they determined the expressions of TETs at mRNA and protein levels by reverse-transcription quantitative polymerase chain reaction (RT-qPCR) and IHC, respectively, and measured 8-oxo-7,8-dihydro-2′-deoxyguanosine (8-oxodG), as a marker of oxidative stress [167], in the samples. Using a highly specific and sensitive isotope-dilution automated online two-dimensional ultra-performance liquid chromatography with tandem mass spectrometry (2D-UPLC-MS/MS), they detected significantly lower levels of 5-hmC in CRC, followed by AD and IDB specimens as compared to normal colonic tissues. To find the relationship between 5-hmC loss and tumor progression, the levels of 5-hmC in CRC samples were correlated to their corresponding tumor stages, from ‘A’ to ‘D’. The significant decrease in 5-hmC content was characteristic for early-stage tumors (stage A), whereas no further reduction in 5-hmC levels was observed in advanced-stage tumors as the disease progressed. Quantification of 8-oxodG by 2D-UPLC-MS/MS showed significantly increased levels of this lesion in IBD and AD samples as compared to both CRC and normal colonic tissues. The elevated levels of 8-oxodG in both IBD and AD [154], which are considered precursor conditions for CRC [203,204,205], are consistent with the proposed role of oxidative stress in the initiation of carcinogenesis [161,162,163,164,165,166,167,168]. Additionally, 8-oxodG may serve as a demethylation signal whereby the base excision repair enzyme, 8-oxoguanine DNA glycosylase (OGG1), bound to this lesion can recruit TET1, which in turn promotes DNA demethylation in response to DNA damage caused by oxidative stress [206]. This may, at least, partially explain the significant reductions in 5-hmC levels concomitant with the increased 8-oxodG levels found in AD and IBD samples as compared to normal colonic tissues. Moreover, the expressions of both *TET1* and *TET2* mRNA in CRC and AD samples were significantly lower than those in normal colonic tissues. Reduced expressions of TET1 and TET2 proteins (*p* = 0.003 and *p* = 0.06, respectively) in CRC than normal colonic tissues were also confirmed by IHC analysis. No changes in expression of TET3 at mRNA or protein level were observed in the analyzed samples [154]. 

Chapman et al. [207] investigated whether 5-hmC plays a role in the regulation of differentiation in colonocytes. The authors measured changes in the levels and genomic distribution of 5-hmC in T84 colon adenocarcinoma cells during cell–cell adhesion-initiated differentiation. They demonstrated that total 5-hmC levels increase during T84 cell differentiation, as determined by immunodot blot assay. Mapping of 5-hmC in the genomic DNA of cells at increasing intervals during differentiation (days 0, 4, 12, and 15) by the hMe-Seal method revealed progressive enrichment of 5-hmC at CpG islands, CpG shores, promoters, and gene bodies. KEGG pathway analysis showed that 5-hmC enriched regions localize to genes involved in epithelial barrier function, including focal adhesion, adherens junctions, regulation of actin cytoskeleton, and endocytosis. Homer motif analysis of regions with 5-hmC peaks predicted that they bind the HNF4A, RXRA, and CDX2 transcription factors, which are known to regulate intestinal development. Examination of HNF4A binding sites of *VAV2* and *GNA12* by TET-assisted bisulfite sequencing (TAB-seq) confirmed that the gain of 5-hmC is accompanied by demethylation at the binding sites of these oncogenes. As HNF4A binding sites have been associated with regions losing methylation in intestinal differentiation [208], the observed formation of 5-hmC at these sites may provide a mechanism for this association. RNA-seq and KEGG pathway analyses showed that genes associated with the mitogen-activated protein kinase (MAPK) signaling pathway are induced over the course of differentiation in T84 cells, whilst numerous metabolic and disease-associated pathways are repressed, simultaneously. The upregulated genes show higher 5-hmC content than genes that are downregulated or exhibit unchanged expression over the time course of differentiation. A reverse analysis examining expression as a function of 5-hmC further confirmed that highly hydroxymethylated genes are more overexpressed and genes with the highest levels of 5-hmC are more likely to be induced and have a greater median fold change than genes with lower 5-hmC levels. The observed association of 5-hmC with highly expressed and induced genes suggests that 5-hmC has an important role in the regulation of gene expression during differentiation of colonocytes. Furthermore, the authors showed that TET1 expression is induced during the time course of differentiation in T84 cells, and *TET1* knockdown alters the expression of genes coding for proteins targeted to the cell membrane and extracellular space, thus inhibiting barrier formation of colonocytes. Comparison of genomic regions covered by 5-hmC in differentiated T84 cells in vitro and primary human colonocytes (*n* = 2) revealed a similar pattern of distribution, whilst a direct correlation was found between gene-specific 5-hmC changes and alterations in gene expression in human colon cancer tissues [207].

Li et al. [209] performed genome-wide profiling of 5-hmC in the genomic DNA (gDNA) of paired tumor and adjacent tissues collected from 80 colorectal cancer patients and in plasma cfDNA samples from patients and healthy controls (*n* = 90). Global 5-hmC levels were markedly decreased (on average an 85% drop) in the gDNA of tumors as compared to adjacent healthy tissues, as determined by ultra-sensitive capillary electrophoresis–electrospray ionization–mass spectrometry. A more limited decrease in global 5-hmC levels was detected in the cfDNA of colorectal cancer patients as compared to healthy controls, consistent with the low proportions of tumor-derived DNA in the total cfDNA pool. In the cfDNA of cancer patients as compared to controls, 5-hmC was enriched within gene bodies and DNase I sensitive sites, whereas it was depleted at TSS, CpG islands, and TF binding sites relative to the flanking regions. This implies that 5-hmC accumulates at positions surrounding TFs at active transcription sites. This was confirmed in the same samples where 5-hmC was enriched in regions marked by permissive histone modifications, such as H3K27ac, H3K4me1, and H3K9me1, whereas it was underrepresented in regions marked by the repressive modification H3K9me3. The average 5-hmC profiles of cfDNA were distinct from those of tissue and white blood cell gDNA, which might be ascribed to the different cells of origin and/or varying extent of DNA degradation in cell-free circulation. Among gDNA 5-hmC profiles, variations attributable to tissue identity (colon tissues vs. white blood cells) were dominant over variations related to disease status (cancer patients vs. healthy individuals and tumors vs. adjacent normal tissues). Presumably, when gDNA from tumor tissue is released into plasma and diluted with the large quantities of background cfDNA derived from various other tissues, the tumor signal detected at a given locus is determined by the order of locus-, tissue-, and disease-specific variations. A model-based classifier using differentially hydroxymethylated loci identified in cfDNA and gDNA was developed, which predicted disease status, with high sensitivity (80–88%) and specificity (83–100%) in independent subpopulations of patients and healthy controls. This classifier also performed better than the conventional biomarkers (including carcinoembryonic antigen (CEA), alpha-fetoprotein (AFP), carbohydrate antigens (CA125, CA15-3, CA19-9, and CA72-4), cytokeratin 19, and neuron-specific enolase (NSE)) and epidemiological risk factors (including indices of overweight, obesity, and alcohol consumption and previous history of cancer) when predicting colorectal cancer. RNA-seq analysis of two tumors from colorectal cancer patients and paired adjacent tissues showed a significant correlation between changes in gene expression levels and alterations in 5-hmC levels in gene bodies in tumor samples (*p* = 9.8 × 10^6^). DAVID pathway analysis revealed that genes with altered 5-hmC levels in tumor gDNA or cancer cfDNA were enriched in cancer- and metastasis-related pathways [209].

Rawluzko-Wieczorek et al. [210] investigated alterations in mRNA levels and promoter methylation of *TET1*, *TET2,* and *TET3* in primary cancerous and histopathologically unchanged colorectal tissues from a cohort of 113 patients. Quantitative RT-PCR analysis showed significantly reduced *TET1*, *TET2,* and *TET3* transcript levels in cancerous tissues as compared to histopathologically unchanged tissues. Importantly, patients with high *TET2* mRNA levels in histopathologically unchanged tissues had favorable overall survival and disease-free survival outcomes. This implies that *TET2* mRNA levels may have prognostic potential for CRC patients’ relapse and survival. Furthermore, bisulfite sequencing confirmed promoter hypermethylation in *TET1* in cancerous tissues as compared to histopathologically unchanged tissues in a small portion of patients (12/113 = 10.6%). No DNA methylation was detected in cancerous or histopathologically unchanged tissues at the *TET2* and *TET3* promoters [210]. 

Neri et al. [211] analyzed the levels of *TET1* mRNA and 5-hmC in eight pairs of primary colon cancers and adjacent healthy tissues. Strong reduction in both *TET1* transcript and 5-hmC levels was detected in cancer tissues as compared to controls by RT-qPCR and immunodot blot assays, respectively. Downregulation of *TET1* was independent of tumor stage and the histopathological grade. The authors confirmed this finding by analyzing a metadata set of colon, breast, lung, and rectum primary tumors of varying stages (stages I to IV). Analysis of the genes differentially expressed in this cohort of 887 adenocarcinomas revealed that *TET1*, but not *TET2* or *TET3*, was strongly downregulated in tumors since stage I. This indicates that *TET1* downregulation is an early event in colon tumorigenesis. Additional experiments showed that *TET1* expression and 5-hmC levels were easily detectable in human colon tissues and in normal epithelial colon cells (CCD), whereas no detectable levels of *TET1* transcript or 5-hmC were observed in five different colorectal cancer cell lines (Colo205, HCT116, HT29, SW48, and Caco-2). Silencing of *TET1* in the normal CCD cells using two different shRNAs resulted in increased cell proliferation, suggesting that TET1 has a role in the control of cell growth. Rescuing experiments showed that re-expression of *TET1* in the *TET*-silenced cells induced a full recovery of 5-hmC level and reduced cell proliferation rate. To demonstrate the effects of *TET1* re-expression on the growth of colon cancer cells, Caco-2 and SW48 human colorectal carcinoma cell lines stably expressing *TET1* under the control of a doxycycline (DOX)-inducible promoter were generated. Of note, re-expression of *TET1* did not alter *TET2* or *TET3* expression in these cells. Upon treatment of the cells with DOX, elevation of 5-hmC levels concomitant with a strong reduction in growth rates were observed in both cell lines. These effects were due to the enzymatic activity of *TET1*, as the catalytically dead mutant (hydroxylase-deficient mutant TET1-H1672Y/D1674A) was not able to interfere with cell growth in vitro. To investigate whether *TET1* plays a role in tumor growth in vivo, nude mice were injected with Caco-2 and SW48 cell lines and subsequently treated with DOX to induce *TET1* expression. The size and weight of xenografts expressing *TET1* were markedly smaller than those in the control group, wherein *TET1* was not induced. Growth of the tumor xenografts was blocked not only when *TET1* was induced early after the tumor cells’ inoculation (with both cell lines) but also several days afterwards when the tumors were already established. Functional experiments showed that TET1 inhibits cancer cells’ growth by repressing the WNT signaling pathway via demethylation of the promoters of the WNT inhibitors, Dickkopf Homolog 3 (DKK3) and Dickkopf Homolog 4 (DKK4), as reflected by the increase in 5-hmC and decrease in 5-mC levels in promoters of the respective genes in DOX-treated Caco-2 and SW48 cells as compared to controls. Rescue experiments in colon cancer cell lines in which the *DKK3* and *DKK4* genes were silenced and *TET1* was re-expressed showed that knockdown of both genes restored the cell growth inhibition by *TET1* expression [211]. Collectively, these data indicate that DNA hydroxymethylation mediated by TET1 controlling the WNT signaling is a key player of tumor growth in colon cancer. *TET1* reactivation, although challenging, may represent a novel therapeutic approach for colon cancer and other types of malignancy.

**Table 1:** Summary results of selected studies on DNA hydroxymethylation and three major smoking-associated cancers (i.e., lung, bladder, and colorectal cancers).

Study	Cancer Type	Samples	Method(s)	Key Findings	Ref.
Li et al.	Lung	Tumors from lung cancer patients and adjacent normal tissues (*n* = 3)	oxBS-seq and RNA-seq	- 5-hmC was significantly enriched in promoters, gene bodies, and transcription termination regions. - There was strong positive correlation between 5-hmC and gene expression levels. - The genomic distribution of 5-hmC highly corresponded with the active histone mark H3K4me1.	[178]
Wang et al.	Lung	Lung squamous cell carcinomas and adjacent normal tissues (*n* = 8)	TAB-EPIC	- Global loss of 5-hmC together with enrichment of 5-hmC in CpG islands and gene upstreams was detected in tumors as compared to normal tissues. - The differentially hydroxymethylated genes converged at pathways involved in cellular process, biological regulation, and metabolic process.	[179]
Song et al.	Lung	Plasma cfDNA from lung cancer patients (*n* = 15) and healthy controls (*n* = 8)	hMe-Seal	- Depletion of global 5-hmC levels was detected in cfDNA of lung cancer patients as compared to healthy controls. - The extent of reduction in global 5-hmC levels in patients’ cfDNA increased progressively as the disease advanced from early-stage non-metastatic to late-stage metastatic lung cancer.	[180]
Zhang et al.	Lung	Plasma cfDNA from non-small-cell lung cancer patients (*n* = 66) and healthy controls (*n* = 67)	hMe-Seal	- Significantly increased 5-hmC levels were found in gene bodies and promoter regions in cfDNA of lung cancer patients as compared to controls.	[181]
Forloni et al.	Lung	Lung adenocarcinoma cell lines	shRNA knockdown assays, soft agar assay, and tumorigenicity in nude mice	- Oncogenic EGFR was shown to silence multiple tumor suppressors in lung cancer cell lines via transcriptional downregulation of *TET1* by the C/EBPα transcription factor.	[182]
Peng et al.	Bladder	Bladder tumors and matching normal tissues from urothelial carcinoma patients (*n* = 135) andbladder cancer cell lines and controls	hMeDIP-seq, IHC, IDB, MTS cell proliferation assay, apoptosis assay, colony formation assay, and xenograft mouse tumorigenicity	- Global loss of 5-hmC was detected in tumor tissues from bladder cancer patients as well as bladder cancer cell lines as compared to controls. - 5-hmC levels were significantly decreased within genes or in the regions 2 kb up- or downstream of the genes in bladder tumors as compared to controls. - Depletion of global 5-hmC levels in bladder cancer patients correlated with higher tumor stage, lymphatic metastasis, and shorter overall survival. - The differentially hydroxymethylated genes converged on molecular pathways involved in cancer. - In vitro treatment of bladder cancer cell lines with vitamin C resulted in increased 5-hmC levels and inhibition of malignant phenotypes. - In vivo treatment of mice with vitamin C by *i.p.* injection resulted in increased 5-hmC levels, reduced tumor growth, and decreased tumor burden.	[195]
Munari et al.	Bladder	Tumors and adjacent benign tissues from patients with urothelial cell carcinoma of the bladder (*n* = 55)	IHC	- Global 5-hmC levels were significantly reduced in tumors from patients as compared to control tissues. - The reduction in global 5-hmC levels was not different between superficial tumors and invasive tumors. - The extent of reduction in global 5-hmC levels was not correlated to tumor grade or stage, or patients’ prognosis.	[196]
Zhu et al.	Bladder	Bladder cancer cell lines	shRNA knockdown assays, rescue experiments for gene expression, MSP, RIP, RT-PCR, Western blot, cell migration, invasion, and lung metastasis assays	- The TET1/USP28/CD44/RhoGDIβ pathway was identified as the regulator of the oncogenic activities of *ATG7* for stem-like property, invasion, and lung metastasis of human bladder cancer cells.	[197]
Hu et al.	Bladder	Bladder cancer cell lines	RIP, ChIP, RT-qPCR, Western blot, and cell proliferation, migration, and invasion assays	- The XIST-TET1-p53 regulatory network was identified as a regulator of cell proliferation, migration, and apoptosis in bladder cancer cells.	[198]
Dziaman et al.	Colorectal	Tumors and adjacent normal colonic tissues from patients with CRC (*n* = 97), colon samples from AD (*n* = 39), and IBD patients (*n* = 49)	2D-UPLC-MS/MS, RT-qPCR, and IHC	- 5-hmC levels were significantly lower in tumor tissues from CRC patients, followed by samples from AD and IDB patients as compared to normal colonic tissues. - Whereas early stage (‘A’) tumors had significant reduction in 5-hmC content, no further decrease in 5-hmC levels was found in advanced stage tumors (‘B–D’). - *TET1* and *TET2* mRNA expressions were significantly decreased in CRC and AD samples as compared to normal colon samples. - Reduced expressions of TET1 and TET2 proteins were observed in CRC samples as compared to normal colonic tissues. - No changes in expression of TET3 at mRNA or protein level were observed in the analyzed samples. - The levels of biomarker of oxidative DNA damage (8-oxodG) were significantly increased in samples from IBD and AD patients as compared to samples from CRC patients and normal colonic tissues.	[154]
Chapman et al.	Colorectal	Colon adenocarcinoma cell lines and primary human colonocytes	hMe-Seal, IDB, TAB-seq, RNA-seq, and shRNA knockdown assays	- Global 5-hmC levels increased during differentiation of colon cancer cells. - 5-hmC levels progressively increased at CpG islands, CpG shores, promoters, and gene bodies in colon cancer cells during differentiation (days 0, 4, 12, and 15). - The 5-hmC enriched regions during differentiation of colon cancer cells localized to genes involved in epithelial barrier function (i.e., focal adhesion, adherens junctions, regulation of actin cytoskeleton, and endocytosis). - Genes associated with MAPK signaling pathway were induced during differentiation of colon cancer cells, whereas numerous metabolic and disease-associated pathways were repressed, simultaneously. - The upregulated genes had higher 5-hmC content than genes that were downregulated or exhibited unchanged expression during differentiation of colon cancer cells. - TET1 expression was induced during differentiation of colon cancer cells. - *TET1* knockdown in colonocytes changed the expression of genes coding for proteins targeted to cell membrane and extracellular space, thus inhibiting barrier formation. - Gene-specific 5-hmC changes were directly correlated to expression changes in the corresponding genes in colon cancer tissues.	[207]
Li et al.	Colorectal	Tumors and adjacent normal tissues and plasma cfDNAs from CRC patients (*n* = 80) and plasma cfDNAs from healthy controls (*n* = 90)	CE–ESI–MS and RNA-seq	- Global loss of 5-hmC levels was detected in both tumor tissues and cfDNA from CRC patients, with the former showing more pronounced reduction in 5-hmC content. - 5-hmC was enriched within gene bodies and DNase I sensitive sites in cfDNA of cancer patients as compared to controls, whereas it was depleted at TSS, CpG islands, and TF binding sites relative to the flanking regions. The 5-hmC-enriched regions were marked by permissive histone modifications (H3K27ac, H3K4me1, and H3K9me1), whereas the depleted 5-hmC regions were marked by the repressive modification H3K9me3. - In tumor samples, changes in 5-hmC levels in gene bodies were significantly corelated to expression changes in the corresponding genes. - The differentially hydroxymethylated genes in tumors or cfDNA from CRC patients were enriched in cancer- and metastasis-related pathways. - A classifier, derived from differentially hydroxymethylated loci in cfDNA and gDNA in CRC patients, predicted disease status, with high sensitivity (80–88%) and specificity (83–100%) in independent subpopulations of CRC patients and healthy controls. This classifier also performed better than the conventional biomarkers and epidemiological risk factors when predicting colorectal cancer.	[209]
Rawluzko-Wieczorek et al.	Colorectal	Primary tumors and histopathologically unchanged tissues from CRC patients (*n* = 113)	RT-qPCR and bisulfite sequencing	- *TET1*, *TET2*, and *TET3* transcript levels were significantly reduced in tumors as compared to control tissues. - High *TET2* mRNA levels in histopathologically normal tissues from CRC patients associated with favorable overall survival and disease-free survival. - In tumor samples, promoter hypermethylation was found only in *TET1* (12/113 = 10.6%) but not in *TET2* or *TET3*.	[210]
Neri et al.	Colorectal	Primary tumors and adjacent healthy tissues from colon cancer patients (*n* = 8) and normal epithelial colon cells and CRC cell lines	IDB, RT-qPCR, shRNA knockdown assays, and xenograft mouse tumorigenicity	- *TET1* transcript and 5-hmC levels were both reduced in tumor tissues as compared to controls. - Downregulation of *TET1* was independent of patients’ tumor stage and histopathological grade. - No detectable levels of *TET1* expression or 5-hmC were found in CRC cells lines. - Silencing of *TET1* in normal epithelial colon cells resulted in increased cell proliferation, whereas re-expression of *TET1* in colon cancer cells markedly increased 5-hmC levels and suppressed growth rate. - Mice injected with DOX-induced *TET1-*expressing colon cancer cells developed significantly smaller tumors (both in size and weight) than counterpart mice injected with non-induced *TET1* cells. - Functional studies identified DNA hydroxymethylation mediated by TET1-controlled WNT signaling as a key player of tumor growth in colon cancer.	[211]

Given the extensive body of literature, we have selected illustrative examples of studies on lung, bladder, and colorectal cancers, which are three major smoking-associated cancers. oxBS-seq = oxidative bisulfite sequencing; TAB-EPIC = TET-assisted bisulfite–Infinium Methylation EPIC BeadChip array; cfDNA = cell-free DNA; hMe-Seal = selective chemical labeling of 5-hmC with biotin for genome-wide detection; shRNA = small/short hairpin RNA; EGFR = epidermal growth factor receptor; IHC = immunohistochemistry; IDB = immunodot blot assays; hMeDIP-seq = hydroxymethylated DNA immunoprecipitation with deep sequencing; MSP = methylation-specific polymerase chain reaction; RIP = RNA immunoprecipitation; RT-qPCR = reverse-transcription quantitative polymerase chain reaction; ATG7 = autophagy-related gene 7; ChIP = chromatin immunoprecipitation; XIST = X-inactive specific transcript; CRC = colorectal cancer; AD = benign polyps/colon adenomas; IBD = inflammatory bowel disease; 2D-UPLC-MS/MS = two-dimensional ultra-performance liquid chromatography with tandem mass spectrometry; 8-oxodG = 8-oxo-7,8-dihydro-2′-deoxyguanosine; TAB-seq = TET-assisted bisulfite sequencing; MAPK = mitogen-activated protein kinase; CE–ESI–MS = capillary electrophoresis–electrospray ionization–mass spectrometry; DOX = doxycycline.

## 9. Concluding Remarks and Future Perspectives

In 2009, the remarkable rebirth of an old epigenetic mark, discovered 37 years earlier [31], untangled the much-anticipated mechanism of active DNA demethylation in mammalian cells [32,33]. Since then, a tremendous body of research has implicated DNA hydroxymethylation in a wide variety of biological processes, from pluripotency and cell differentiation to neural system development and aging to an array of diseases, especially cancer [21,22,24,33]. Because smoking is a major risk factor for many types of human cancer [4,6], elucidating the role of DNA hydroxymethylation in smoking-associated carcinogenesis has the potential to translate to biomarker discovery in patient populations as well as in healthy smokers susceptible to cancer. The existing data show a highly complex and interconnected interplay between 5-hmC and TETs in transcriptional dysregulation of key genes involved in tumorigenesis and carcinogenesis, as shown in samples from cancer patients and experimental model systems [21,33,38,114,118,119].

Global depletion of 5-hmC together with locus-specific gain/loss of hydroxymethylation and variation in TETs activity and function, which are highly cell-type dependent, are hallmarks of cancer, as observed in solid tumors from patients with smoking-associated malignancies and in in vitro or in vivo systems [22,23,24]. The detectability of these changes in early stages of carcinogenesis suggests that these alterations may serve as novel biomarkers for the early detection of smoking-associated cancers. The prognostic value of these modifications, however, has yet to be consistently confirmed in cancer patients as the disease progresses and tumor grade and stage advance. 

An area of interest is the use of non- or minimally invasive surrogate tissues, which would faithfully inform the occurrence of epigenetic changes in target organs of smoking-associated cancers. In addition to conventional surrogate tissues, such as peripheral blood, oral epithelial cells, and urine [169,212,213], the use of liquid biopsy to collect body fluid samples containing circulating tumor cells, circulating tumor DNA, and exosomes (i.e., extracellular vesicles filled with DNA, RNA, or proteins) in the blood have been exploited for cancer epigenetic studies [214,215]. These non- or minimally invasive sampling techniques may prove ideal for patient monitoring and population-based studies. So far, analysis of samples collected by these techniques has yielded both promising and inconclusive results. However, caution must be taken in interpreting these results, especially given the cell-type specificity of epigenetic changes [24,214,216]. This is of paramount importance when analyzing liquid biopsy specimens that contain cfDNA, cells, or exosomes that originated from various tissues that enter the blood circulation [214,215,216].

As we continue to investigate the role of DNA hydroxymethylation in the initiation and progression of smoking-associated cancers in patient populations, we should also expand our studies to healthy populations of smokers and nonsmokers. The latter studies, particularly with a prospective design, should facilitate identification and validation of diagnostic and prognostic biomarkers of smoking-associated cancers. With the same token, the growing population of electronic nicotine delivery system (ENDS) users, whose alternative tobacco products are known to contain toxic and carcinogenic compounds (albeit generally at substantially lower levels than tobacco cigarettes) [217,218,219,220,221,222,223], represents yet another important source population.

## Data Availability

The data presented in this review article are contained within the article.

## Abbreviations

2D-UPLC-MS/MS, two-dimensional ultra-performance liquid chromatography with tandem mass spectrometry; 2-HG, 2-hydroxyglutarate; 5-caC, 5-carboxylcytosine; 5-fC, 5-formylcytosine; 5-hmC, 5-hydroxymethylcytosine; 5-mC, 5-methylcytosine; 8-oxodG, 8-oxo-7,8-dihydro-2′-deoxyguanosine; AD, adenomas; AFP, alpha-fetoprotein; α-KG, α-ketoglutarate; ATG7, autophagy-related gene 7; AUF1, AU-rich element RNA-binding protein 1; BER, base excision repair; CA, carbohydrate antigen; CD44s, CD44 standard; CEA, carcinoembryonic antigen; cfDNA, cell-free DNA; CNS, central nervous system; CRC, colorectal cancer; C, cytosine; DKK, 3Dickkopf Homolog; DNMTs, DNA methyltransferases; DHHA, 4,5-dihydroxyhexanoic acid; DOX, doxycycline; EGFR, epidermal growth factor receptor; ENDS, electronic nicotine delivery systems; FH, fumarate hydratase; GO, gene ontology; HIF, hypoxia-inducible factor; IBD, inflammatory bowel disease; IHC, immunohistochemistry; IARC, International Agency for Research on Cancer; IDH, isocitrate dehydrogenase; gDNA, genomic DNA; KEGG, Kyoto Encyclopedia of Genes and Genomes; LUSC, lung squamous cell carcinoma; MAPK, mitogen-activated protein kinase; miRNA, microRNA; NSCLC, non-small-cell lung cancer; NSE, neuron-specific enolase; oxBS-seq, oxidative bisulfite sequencing; OGG1, 8-oxoguanine DNA glycosylase; PRC2, polycomb repressive complex 2; ROS, reactive oxygen species; RT-qPCR, reverse-transcription quantitative polymerase chain reaction; SDH, succinate dehydrogenase; shRNA, small/short hairpin RNA; TAB-seq, TET-assisted bisulfite sequencing; TDG, thymine DNA glycosylase; TET, ten-eleven translocation; TF, transcription factor; TSS, transcription start site; UHRF1, ubiquitin-like with PHD and ring finger domains 1; USP28, ubiquitin specific peptidase 28; XIST, X-inactive specific transcript.
